# Antibiotic Resistance Pattern of Different *Escherichia coli* Phylogenetic Groups Isolated from Human Urinary Tract Infection and Avian Colibacillosis

**DOI:** 10.6091/ibj.1394.2014

**Published:** 2014-10

**Authors:** Ali Kazemnia, Malahat Ahmadi, Mahdi Dilmaghani

**Affiliations:** 1 *Dept. of Microbiology, Faculty of Veterinary Medicine, Urmia University, Iran; *; 2 *Dept. of Cellular and Molecular Biology, West Azerbaijan Veterinary Laboratory, Urmia, Iran*

**Keywords:** *Escherichia coli*, Avian colibacillosis, Phylogenetic grouping

## Abstract

**Background** **: **The emergence and propagation of different phylogenetic groups of antimicrobial-resistant *E. coli* have become a worldwide health concern in human and veterinary medicine. Therefore, the evaluation of the phylogenetic distribution of antibiotic-resistant *E. coli* is important for therapeutic and economic purposes. The aims of this study were to determine phylogenetic groups and patterns of antibiotic resistance of *E. coli* strains isolated from human urinary tract infection and avian colibacillosis. **Methods: **A total of 50 *E. coli* isolates (25 from human urinary tract infection and 25 from avian colibacillosis) were characterized by culture and assigned as different phylogenetic groups (A, B1, B2, and D) by triplex PCR assay. Kirby-Bauer disk diffusion method was used to assess the susceptibility of all isolates to ten antibiotics. **Results: **Results showed that the majority of the human and poultry isolates belonged to phylogenetic groups A and B2 and phylogenetic group B1 of the avian pathogenic strain isolates were the most drug-resistant isolates. Most of the isolates were resistant to at least five antibiotics, and multiple drug resistance was observed in 98% of *E. coli* isolates. A high degree of resistance was seen against penicillin and erythromycin. **Conclusion: **According to the results of this study, multidrug-resistance among isolates and high relation between phylogenetic groups and resistance in both human and poultry isolates were observed.

## INTRODUCTION

*E. coli* is well known for its capacity to cause a variety of infections. In addition to gastrointestinal illness typically manifested as diarrhea, *E. coli* also causes a variety of diseases outside the intestinal tracts of humans and animals, which include urinary tract infections, meningitis, sepsis, abdominal infections, osteomyelitis, cellulitis, wound infections, and colibacillosis [1, 2]. 

Colibacillosis is one of the most frequently reported diseases in the poultry industry. This disease is economically relevant to poultry producers, because it causes high mortality and poor egg quality in broilers and laying hen flocks, respectively. Especially on rural farms, *E. coli* infections seriously affect production and bird survival, since biosecurity and hygiene are frequently unheeded. The disease can be controlled using antimicrobials for therapy and prophylaxis [3]. On rural farms, increase in antimicrobial resistance in developing countries has become a major concern due to frequent use of antibiotics, which promotes multiple drug resistance (MDR) in urinary pathogenic *E. coli* (UPEC) in both veterinary and human medicine [4, 5]. *E. coli* is responsible for up to 90% of all community-acquired and almost 50% of nosocomial urinary tract infections. β-lactam and quinolone antimicrobials are the most frequently prescribed drugs for treatment in clinical settings [5]. Transfer of antimicrobial-resistant strains of *E. coli* to the food chain from poultry source is a well-recognized phenomenon. The avian pathogenic strains (APEC), which cause cellulitis, septicemia and colibacillosis in poultry, may link to extra-intestinal pathogenic *E. coli* strains in humans. These extra-intestinal pathogenic *E. coli* possess some virulence factors that enable them to cause disease outside the intestinal tract. Therefore, the resistant APEC may transfer antimicrobial-resistant strains to human via the food chain and can have implications for treatment of urinary tract and other extra-intestinal infections. This matter may have effect on treatment of salmonellosis and other enteric infections as well. This occurrence of any changes in the resistance profile of avian strains of *E. coli* should be mentioned and evaluated [2].

Four main phylogenetic groups have been shown in *E. coli*, including phylogenetic groups of A, B1, B2, and D. Phylogenetic grouping can carried out by multilocus enzyme electrophoresis, ribotyping or patterns of the strains in the *E. coli* reference collection, but these reference techniques are complex and time-consuming and also require a collection of typed strains [6, 7]. Clermont *et al.* [6] described a rapid technique for determining the phylogenetic groups of *E. coli* strains based on PCR detection of the *chuA* and *yjaA* genes and DNA fragment TspE4.C2. The virulent extra-intestinal strains belong mainly to group B2 and, to a lesser extent, to group D, whereas most commensal strains belong to groups A and B1 [6]. Thus, according to the importance of different *E. coli* phylogenetic groups and the role of its antibiotic resistance pattern, the purposes of this study were as follow: 1) to determine different phylogenetic groups of isolated *E. coli*; 2) to determine antibiotic resistance profile of isolated *E. coli*, and 3) to determine any correlation between different phylogenetic groups and antibiotic resistance of isolated* E. coli*.

## MATERIALS AND METHODS

***Sample collection. ***From January to November 2012, a total of 235 samples including 91 urine samples from hospitalized patients and 144 samples from poultry carcasses suspected to colibacillosis were collected.

***Isolation of Escherichia coli. ***Samples were cultured on McConkey agar (Merck, Germany) and eosin methylen blue agar agar plates (Merck, Germany) and incubated at 37°C for 24 hours. Suspected *E. coli* colonies were identified by standard methods based on colonial appearance, and bacterial morphology, followed by biochemical characteristics. Pure colonies, which were urease negative, indole positive, oxidase negative, citrate negative, motility positive, methyl red positive, Voges-Proskauer negative, and lactose fermentation positive on triple sugar iron agar were identified as *E. coli *[2]. 

***Antibiotic susceptibility testing. ***The antibacterial susceptibility testing of all *E. coli* isolates was performed using the Kirby-Bauer disk diffusion method. A volume of 100 µl of an overnight growth of each *E. coli* isolate on Mueller-Hinton broth with 0.5 McFarland standard turbidity was streaked on Mueller-Hinton agar plates. The routinely used 10 antibiotic discs, all from HiMedia^®^ (India), including penicillin, ampicillin, amoxicillin, cefixime, cephalexin, ciprofloxacin, nalidixic acid, erythromycin, tetra-cycline and gentamicin were placed on the surface of the inoculated plates. The plates were incubated at 37°C for 24 hours. The zones of inhibition were measured and compared with standard chart and with *E. coli* ATCC 25922 and *Staphylococcus aureus* ATCC 29213 as antibiotic controls. Isolates with intermediate resistance were defined as susceptible, and the isolates were considered as multidrug resistant if they were resistant to at least three classes of antibiotics [4, 8, 9].

***DNA extraction. ***Two colonies of pure isolated bacteria were placed into a tube containing 100 µl of double distilled water. Tubes were heated at 100ºC for 10 minutes, and then the cells were pelleted by centrifugation. The supernatant containing DNA was taken out and stored at -20ºC [2].

***Multiplex PCR reaction for isolates.*** All* E. coli* isolates tested by multiplex PCR have been described previously [6]. As shown in Table 1, three sets of primers were used in this study, including *ChuA*, *YjaA*, and TspE4C2, which generate 279 bp, 211 bp, and 152 bp fragments, respectively. Multiplex PCR reaction was performed in a 25 µl reaction mixture, containing PCR buffer (10 mM Tris-HCl, 50 mM KCl, and 1.5 mM MgCl_2_, pH 8.7), dNTP (200 μM), each primer (0.4 μM), Taq DNA polymerase (1U), and template DNA (2 µl). PCR reaction was performed in a DNA thermocycler (Model CP2-003; Corbett, Sydney, Australia) as follows: Initial denaturation at 94ºC for 4 min, 30 cycles of denaturation at 94ºC for 5 s, annealing at 59ºC for 10 s, elongation at 72ºC for 30 s and a final extension step of at 72ºC for 5 min, followed by a hold at 4ºC. PCR products were electrophoresed on 1.5% agarose gel containing ethidiumbromide at 80 V for 1 h.

**Table 1:** Primer characteristics used in this study

**Primer**	**Target gene**	**Primer ** **length (bp)**	**Sequence**	**Amplified ** **fragment** **size (bp)**	**References**
ChuA.1	*ChuA*	20	5´-GACGAACCAACGGTCAGGAT-3´	279	[6]
ChuA.2		20	5´-TGCCGCCAGTACCAAAGACA-3´		
					
YjaA.1	*YjaA*	20	5´-TGAAGTGTCAGGAGACGCTG-3´	211	[6]
YjaA.2		21	5´-ATGGAGAATGCGTTCCTCAAC-3´		
					
TspE4C2.1	TspE4C2	20	5´-GAGTAATGTCGGGGCATTCA-3´	152	[6]
TspE4C2.2		20	5´-CGCGCCAACAAAGTATTACG-3´		

**Table 2:** Assignation of different phylogenetic groups

** Gene**	***chuA***	***yjaA***	**TspE4.C2**
**Group**			
Group A	NP*	V**	NP
Group B1	NP	V	P***
Group B2	P	P	V
Group D	P	NP	V

* Not present (group does not possess gene),

** Variable (possession of gene is variable),

*** Present (group possesses gene)

***Determination of different phylogenetic groups. ***Isolates were assigned to one of four groups (A, B1, B2, or D) based on their possession of two genes (chuA and yjaA) and a DNA fragment (TSPE4.C2) (Table 2).

## RESULTS

***Identification of Escherichia coli. ***Out of 91 urine samples from hospitalized patients, 27.47% (n = 25) *E. coli* and out of 144 poultry carcasses samples suspected to colibacillosis, 17.36% (n = 25) *E. coli* were isolated using culture and biochemical tests.

**Fig. 1:**
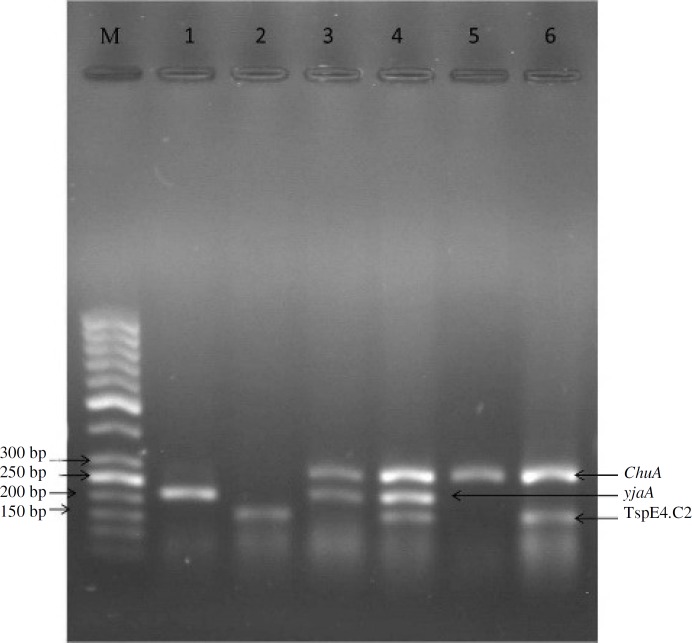
Triplex PCR of isolates. Lane M, (ladder 50 bp, Fermentas); lane 1, group A; lane 2, group B1; lanes 3 and 4, group B2, and lanes 5 and 6, group D

***Phylogenetic grouping of isolates using multiplex PCR. ***a) In UPEC isolates, multiplex PCR (triplex PCR) analysis (Fig. 1) of the 25 isolates revealed that the distribution of different phylogenetic groups among UPEC isolates for groups A, B2, and D were 8 (32%), 

10 (40%), and 7 (28%), respectively. However, phylogenetic group B1 were not detected in UPEC isolates. b) In avian pathogenic strain isolates, the distribution of different phylogenetic groups using multiplex PCR among 25 APEC isolates for groups A, B1, B2, and D were 9 (36%), 4 (16%), 7 (28%), and 5 (20%), respectively (Table 3). c) Among all *E. coli* isolates, the majority belonged to phylogenetic groups A and B2.

***Pattern of antibiotic resistance. ***From 50 tested *E. coli* isolates, all of them (100%) were resistant to penicillin and erythromycin, followed by 49 (98%) to nalidixic acid, 47 (94%) to cephalexin, 43 (86%) to amoxicillin, 42 (84%) to ampicillin, 37 (74%) to ciprofloxacin, 32 (64%) to tetracycline, 27 (54%) to cefixime and 18 (36%) to gentamicin. The results showed that the most effective antibiotic against UPEC isolates was ciprofloxacin (48%) and against APEC isolates was gentamicin (96%). Forty nine (98%) of the MDR isolates were resistant to ≥ 5 antimicrobial medicines. The pattern of drug resistance in different phylogenetic groups of UPEC and APEC is shown in Table 4. In phylogenetic group A, all APEC and UPEC isolates were highly resistant to penicillin, cephalexin, nalidixic acid and tetracycline but not too much to gentamicin. Also, the APEC isolates were much more sensitive to gentamicin as compared to the UPEC isolates. In phylogenetic group B1, APEC isolates were resistant to almost all of the antimicrobial medicines. 

**Table 3:** Number of different phylogenetic groups between UPEC and APEC

** Group**	**A** **No. (%)**	**B1** **No. (%)**	**B2** **No. (%)**	**D** **No. (%)**	**Total**
**Isolates**					
UPEC	8 (32)	0 (0)	10 (40)	7 (28)	25
APEC	9 (36)	4 (16)	7 (28)	5 (20)	25
Total	17 (34)	4 (8)	17 (34)	12 (24)	50

UPEC, urinary pathogenic *E. coli*; APEC, avian pathogenic strains

**Table 4:** Number (%) of resistant and susceptible isolates against different antibiotics among different phylogenetic groups

**Group**		**A**			**B**_1_					**B**_2_			**D**			**Total (%)**
**Type**		**UPEC**	**APEC**		**UPEC**	**APEC**			**UPEC**		**APEC**		**UPEC**	**APEC**		
Number		8	9		0	4			10		7		7	5		50
Gentamicin	R	5	0		-	1			7		0		5	0		18 (36)
	S	3	9		-	3			3		7		2	5		32 (64)
Tetracycline	R	3	6		-	4			7		1		7	4		32 (64)
	S	5	3		-	0			3		6		0	1		18 (36)
Erythromycin	R	8	9		-	4			10		7		7	5		50 (100)
	S	0	0		-	0			0		0		0	0		0 (0)
Ciprofloxacin	R	5	9		-	3			5		7		3	5		37 (74)
	S	3	0		-	1			5		0		4	0		13 (26)
Nalidixic acid	R	7	9		-	4			10		7		7	5		49 (98)
	S	1	0		-	0			0		0		0	0		1 (2)
Cefixime	R	3	1		-	3			8		4		5	3		27 (54)
	S	5	8		-	1			2		3		2	2		23 (46)
Cephalexin	R	8	8		-	4			10		5		7	5		47 (94)
	S	0	1		-	0			0		2		0	0		3 (6)
Amoxicillin	R	4	9		-	4			8		7		7	4		43 (86)
	S	4	0		-	0			2		0		0	1		7 (14)
Ampicillin	R	4	8		-	4			9		5		7	5		42 (84)
	S	4	1		-	0			1		2		0	0		8 (16)
Penicillin	R	8	9		-	4			10		7		7	5		50 (100)
	S	0	0		-	0			0		0		0	0		0 (0)
MDR		7	9		-	4			10		7		7	5		49 (98)

R, resistant; S, susceptible; UPEC, urinary pathogenic *E. coli*; APEC, avian pathogenic strains; MDR, multiple drug resistance

In phylogenetic group B2, all isolates of both APEC and UPEC were resistant to penicillin, erythromycin, and nalidixic acid, and almost all of the isolates were resistant to ampicillin, amoxicillin, and cephalexin. There was a different pattern of sensitivity to gentamicin and tetracycline in the isolates of different sources (UPEC and APEC). In phylogenetic group D, all isolates in both UPEC and APEC were resistant to penicillin, ampicillin, cephalexin, nalidixic acid, and erythromycin, and almost all of them were resistant to amoxicillin, cefixime and tetracycline. Furthermore, 10 (40%) of UPEC isolates belonging to group B2 and 9 (36%) of APEC isolates belonging to group A were the main phylogenetic groups of MDR isolates.

## DISCUSSION

The emergence, propagation, accumulation, and maintenance of strains of antimicrobial-resistant pathogenic bacteria have become a worldwide health concern in human and veterinary medicine. The intensive therapeutic uses and misuses of antimicrobial agents in humans and companion animals as well as their therapeutic, prophylactic, and subtherapeutic uses for growth promotion in food animals have substantially increased selective pressures on both pathogenic and commensal bacteria, thus favoring the propagation, accumulation, and maintenance of antimicrobial-resistant bacteria [10]. 

In the present study, identification of *E. coli* was conducted using standard culture and biochemical tests from hamun urine and avian colibacillosis samples, followed by multiplex PCR to assign each isolate to a certain phylogenetic group (A, B1, B2, and D). According to the recent phylogenetic studies on *E. coli *[1, 4, 11, 12], extra-intestinal pathogenic *E. coli *strains are mostly derived from the B2 phylogenetic group and, to a lesser extent, from group D, which are in line with the results of present study. It has been shown that the most commensal *E. coli* strain belongs to group A [13], and the majority of APEC isolates in this study belongs to group A.

There is a hypothesis that the urovirulent *E. coli* clones, present in the human intestine, come from fecal-oral route, and poultry is a candidate vehicle that transmits *E. coli* from poultry to human [1, 14]. The results obtained from phylogenetic typing in the present study, were not enough to accept or reject this theory. Indeed, further studies based on serogrouping, plasmid-related genes genotyping, and virulence gene genotyping will clarify this hypothesis [15]. In the present study, none of the UPEC isolates belonged to phylogenetic group B1, contrary to some of the previous studies [5, 11, 16]. This controversy could probably be due to the bacterial characteristics in different geographic regions, antibiotics usage or host genetic factors, and the number of isolated *E. coli* in present study.

The present investigation was conducted to achieve resistance profile of clinical isolates from our local area against commonly prescribed antibiotics. The *in vitro* antibiotic susceptibility pattern of the isolates showed high resistance to commonly used antibiotics such as penicillin, erythromycin, nalidixic acid, cephalexin, amoxicillin, ampicillin and ciprofloxacin. Our findings are in agreement with those of previous studies [2, 4, 8, 17, 18]. This high degree of resistance could be explained by the fact that these drugs are easily available without physicians' prescriptions from pharmacy in developing countries. In our study, 100% of the isolates were resistant against at least five antibiotics, which makes them a serious health problem, and 98% of them were MDR, which is close to the prevalence (≥70%) reported in Europe, India, and USA [10, 19-21].

The relation between phylogenetic background and antibiotic resistance showed that all UPEC isolates of group A were both resistant to penicillin and cephalexin. In contrast, all APEC isolates in group A were resistant to penicillin, erythromycin, ciprofloxacin, nalidixic acid, cephalexin, amoxicillin, and ampicillin. These results revealed that treatment of the diseases associated with APEC isolates in group A is much more difficult than UPEC isolates. In group B1 of APEC isolates, all of them were resistant to tetracycline, erythromycin, nalidixic acid, cefixime, cephalexin, penicillin, amoxicillin, and ampicillin. Because of extensive use of antibiotics to promote weight gain and for prophylaxis purposes, this high level of resistance was expected in commensal organisms (group A and B1 of APEC isolates) [22]. 

In group B2 of UPEC isolates, all of them were resistant to erythromycin, nalidixic acid, cephalexin, ampicillin and penicillin, whereas group B2 of APEC isolates were resistant to penicillin, erythromycin, ciprofloxacin, nalidixic acid, and amoxicillin. Among group D of UPEC isolates, all of them were resistant to tetracycline, erythromycin, nalidixic acid, cephalexin, penicillin, amoxicillin and ampicillin. However, in group D of APEC isolates, all of them were resistant to erythromycin, ciprofloxacin, nalidixic acid, cephalexin, amoxicillin, ampicillin, and penicillin. This fact could be due to the high level of prevalence [15, 23, 24], virulence [1, 2, 15, 25], resistance [2, 22], and plasmid-mediated resistance gene transfer in these isolates (groups B2 and D).

The results of this study, contrary to the previous reports [2, 4], revealed that phylogenetic group B1 of the APEC isolates, group D (UPEC and APEC), group A of APEC isolates, group B2 (UPEC and APEC), and group A of UPEC isolates are the most drug-resistant isolates. Evidence suggests that there is a relation between the overuse of antimicrobials, antimicrobial residues in poultry production, and the increasing emergence of resistant bacteria [26-28]. Therefore, careful choice of antibiotics based on the surveillance programs is necessary to avoid treatment failures and to prevent transmission of antimicrobial residues from poultry production to human food chain.
